# Positive and negative facial valence perception are modulated differently by eccentricity in the parafovea: Replication from KDEF to NimStim

**DOI:** 10.1038/s41598-024-63724-2

**Published:** 2024-06-14

**Authors:** Vasilisa Akselevich, Sharon Gilaie-Dotan

**Affiliations:** 1 School of Optometry and Vision Science, Faculty of Life Science, Bar Ilan University, 5290002 Ramat Gan, Israel; 2 The Gonda Multidisciplinary Brain Research Center, Bar Ilan University, Ramat Gan, Israel; 3 UCL Institute of Cognitive Neuroscience, London, UK

**Keywords:** Emotional valence, Face expression, Periphery, Replication study, Emotion perception, Human behaviour, Emotion, Social behaviour, Visual system

## Abstract

While perceiving the emotional state of others may be crucial for our behavior even when this information is present outside of central vision, emotion perception studies typically focus on central visual field. We have recently investigated emotional valence (pleasantness) perception across the parafovea (≤ 4°) and found that for briefly presented (200 ms) emotional face images (from the established KDEF image-set), positive (happy) valence was the least affected by eccentricity (distance from the central visual field) and negative (fearful) valence the most. Furthermore, we found that performance at 2° predicted performance at 4°. Here we tested (n = 37) whether these effects replicate with face stimuli of different identities from a different well-established image-set (NimStim). All our prior findings replicated and eccentricity-based modulation magnitude was smaller with NimStim (~ 16.6% accuracy reduction at 4°) than with KDEF stimuli (~ 27.3% reduction). Our current investigations support our earlier findings that for briefly presented parafoveal stimuli, positive and negative valence perception are differently affected by eccentricity and may be dissociated. Furthermore, our results highlight the importance of investigating emotions beyond central vision and demonstrate commonalities and differences across different image sets in the parafovea, emphasizing the contribution of replication studies to substantiate our knowledge about perceptual mechanisms.

## Introduction

Our perception and behavior are influenced by the emotional state of those around us, even when not reaching our unawareness^1–3^. Visual information related to the emotional state of faces around us (aka facial expressions) plays an important role in allowing us to sense these social cues^4,5^, and these are of great interest in developmental, sociopsychological, and psychopathological fields^4–8^. Facial expressions are classically classified according to the basic emotion they convey (e.g., happiness, anger, fear, disgust^9,10^), according to the pleasantness of that emotion (termed emotional valence and could be positive (e.g. happiness), negative (e.g. fearful), or neutral^11,12^) or by their intensity (which could be low to high^13–15^). While faces and therefore facial expressions can appear at many locations across the visual field, most investigations examining perception of facial expression focus on central rather than peripheral vision^12,14,16^. Studies examining how eccentricity (distance from central visual field) modulates facial expression perception (predominantly expression categorization) typically examine eccentricity effects at peripheral locations right and left of fixation^17–20^. The findings from most of these studies is that performance is highest for emotions of positive valence relative to other emotions (of negative or neutral valence) both in central and in peripheral locations (even up to 30° to the right or left of fixation). In fact, one study finds that the sensitivity for happy emotions (positive valence) is not affected by eccentricity even up to 6° right or left of fixation^20^. Another study investigating facial expression perception in the parafovea (at 3.8°) while presenting stimuli at different locations in the visual field also finds highest performance for expressions of positive valence and lower for negative valence^21^.

We have recently examined in a parametric fashion how eccentricity in the parafovea (≤ 4°) influences facial expression valence perception and found that performance significantly decreased (lower accuracy and slower response times) with growing eccentricity for each valence but importantly, not in the same manner for all valences^22^. Specifically, we found that positive valence was the least affected by eccentricity and negative valence the most (at 4° positive valence accuracy decreased by ~ 19% relative to central vision while negative valence accuracy decreased by ~ 38%). In addition, we found that parafoveal accuracies at 2° and 4° were significantly associated within-valence (i.e. 2° positive to 4° positive, 2° negative to 4° negative and 2° neutral to 4° neutral) but not between valences (e.g. 2° positive to 4° negative, 2° negative to 4° neutral, etc.), suggesting that parafoveal perception of different valences may rely on dissociated mechanisms. In that study we used facial emotional expression stimuli from one well-established image-set (KDEF^23^) using fearful expressions representing negative valence and happy expressions representing positive valence. Most earlier studies investigating facial expression perception in peripheral vision also relied on stimuli from one image-set (as KDEF^17,19–21^ or Ekman^18^). However, parafoveal perception of emotional face expressions may be modulated by the image set being used and in fact differences in performance have been reported when using different image-sets and relying on central vision (e.g. between KDEF and NimStim^24,25^, or between other image sets^13,14^).

While we assumed our recent findings^22^ were not specific to the KDEF stimuli and to the 12 identities used in the study, it is hard to rule out such a potential confound^24^. Furthermore, there are inconsistencies in the field with respect to heightened performance to positive or to negative valence^12,26–28^ (e.g. several studies report on anger superiority such that angry expressions are easier to detect in a crowd^29–32^ whereas other studies report that happy expressions and not angry ones are easier to detect in crowds^27,33,34^). In addition, given the replication crisis in the field of cognitive psychology^35–37^, it is important to examine whether our results replicate with different images from another image set in a different cohort.

Therefore, here, using the same paradigm, we examined whether our results replicate with different emotional stimuli of different identities from a different well-established emotional faces image-set (NimStim)^25^. We assumed that the findings from our earlier study would replicate, and specifically that performance would decrease with eccentricity and that different valences would be modulated differently by eccentricity (but in the same order). To evaluate potential image-set influences on parafoveal facial expression valence perception, we also aimed to directly compare performances measured in the KDEF and in the NimStim studies. Given the recent replication crisis in the field of cognitive psychology^35–37^, we also intended to follow published recommendations that aim to increase transparency, reduce potential latent biases, and support the generalization of our earlier findings. Importantly, as studies comparing between different facial expressions image sets are often based on central vision performance^13,24^ and there are no studies to the best of our knowledge that examined performance differences between emotional image sets beyond central vision, our study would allow examining how image set differences modulate performance in parafoveal vision.

## Methods

### Transparency and openness

The methods, procedures, measurements, and data analysis of the current study follow those used in our recent study^22^ (see details and data at https://osf.io/8t6r2/). The current study’s design and its analyses were not preregistered. The data reported here were collected between March 2022 and January 2023. The data and analysis are available at https://osf.io/25fnw^38^. We report here how we determined our sample size, all data exclusions, and all measures in the study. Statistical analyses were performed with SPSS Statistics version 28.0.1.1 for Windows (IBM Corp. Released 2020).

### Participants

A group of thirty-nine healthy adults (aged 18–35 years, mean age 26 ± 4.9 (SD) years; 19 women, 36 right-handed) with normal or corrected to normal vision (VA =  − 0.09 logMAR ± 0.07 (SD)) participated in our study. Participants’ educational background was heterogeneous (one finishing high school (18 y.o.), 7 had high school diplomas, 13 were higher-education students, and 18 had higher-education diplomas), and more demographics information are available at on OSF at https://osf.io/25fnw/. Two participants were excluded from the analysis since for one participant we had technical problems in recording the responses and the other participant appeared to be looking away from the screen according to the fixation analysis during most of the experiment (fixating only in 27.5% of all trials). Two additional participants partially completed the experiment (one a third of the trials (NS18, completed 216 of 648 trials), one ~ half of the trials (NS23, completed 324 of 648 trials)) and their data were included in the study since their accuracy at central vision (0°) in all of the conditions was consistent with our inclusion criterion (above floor performance (set at chance level, as in our earlier study^22^) for all trials before we applied the fixation-based exclusion criteria see more details at OSF at https://osf.io/25fnw/). All recruited participants were reimbursed for their efforts. The experimental protocol was approved by the Bar-Ilan University ethics committee and in accordance with the Declaration of Helsinki. Participants gave written informed consent to take part in the study prior to their participation.

#### Sample size estimation

The sample size in our previous study^22^ was planned based on an earlier study that investigated eccentricity effects on face perception at foveal to parafoveal locations^39^ and on additional studies focusing on perception of emotional faces in the parafovea (Bayle et al.,^40^, with n = 20; Rigoulot et al.^41^, with n = 16). For the current study we conducted an a-priory sample size estimation for eccentricity and for valence using G*Power version 3.1.9.7^42^ based on data from our previously published study that examined eccentricity and valence effects on emotion perception (Akselevich & Gilaie-Dotan^22^, n = 37; the effect sizes and achieved power are reported in Supplementary Material Table S1 at https://osf.io/25fnw^38^). This sample size estimation suggested that 15(6) participants were required for detecting similar valence(eccentricity) effect with *η*_*p*_^*2*^ = 0.355(*η*_*p*_^*2*^ = 0.853) at significance criterion of *α* = 0.05 and *β* = 0.8 (for *α* = 0.005 and *β* = 0.8: n = 24(9), and for *α* = 0.005 and *β* = 0.9: n = 27(9)). Based on these estimations, and since we were interested in comparing the results of the current study to those of our previous study, we collected data that would allow the analysis to be performed on a cohort of the same size as that in our previous study (single trial experiment, n = 37). The effect sizes and achieved power of the current study are reported in Supplementary Material Table S2 at https://osf.io/25fnw^38^.

### Stimuli and apparatus

**Figure 1:**
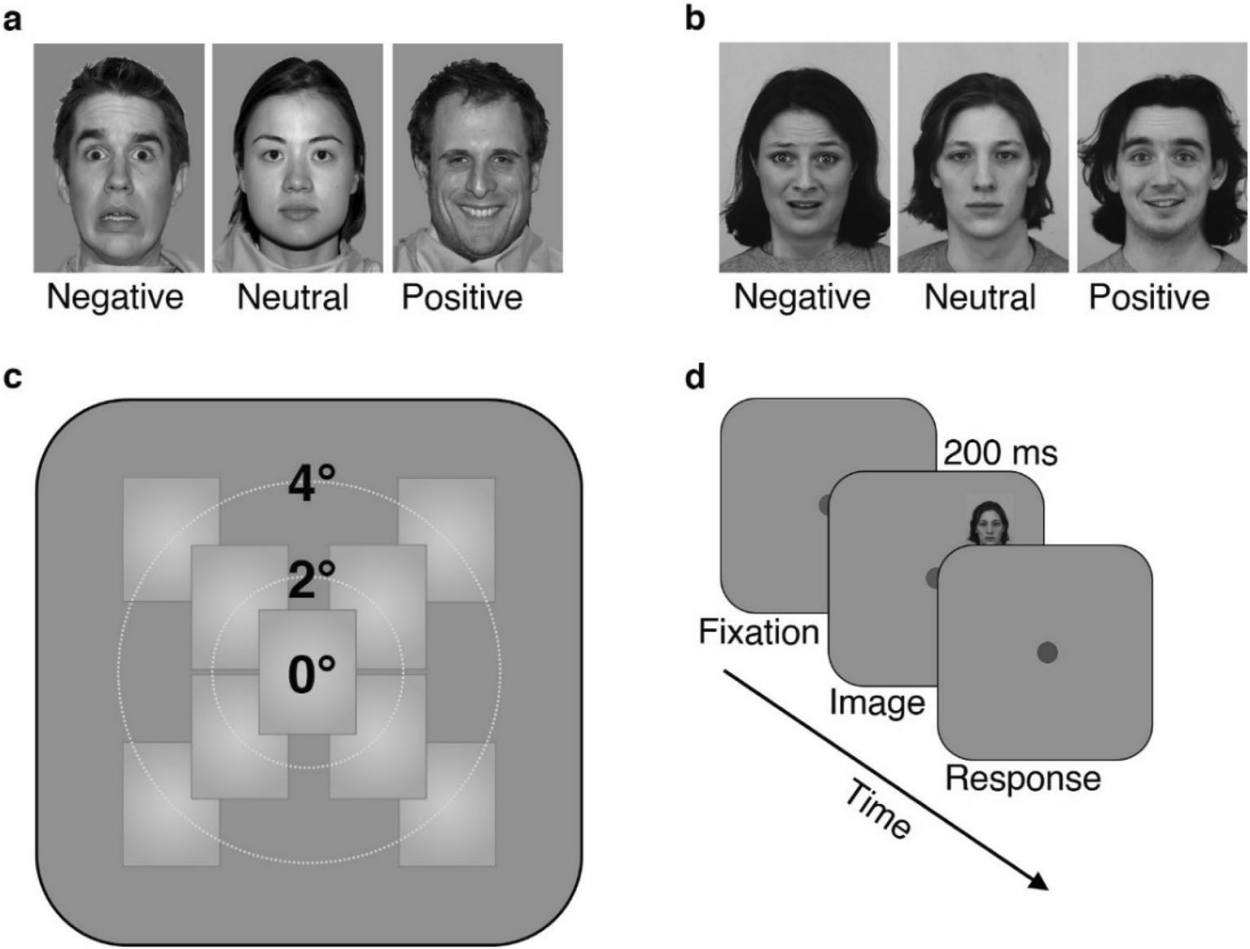
Experimental design. (**a**) NimStim image-set^25^ representative stimuli used in the current study with negative (fearful), neutral, or positive (happy) valence. The de-identified NimStim image IDs in this figure are 28m_fe_o, 18f_ne_c, 21m_ha_o. (**b**) KDEF^23^ image-set representative stimuli used in our previous study^22^; in this figure the de-identified KDEF image IDs are AF01AFS, AM18NES and AM35HAS. (**c**) The 9 possible stimulus locations at central (0°) or parafoveal locations (at one of the quadrants at 2° or 4°). (**d**) Example of experimental trial timeline. Each trial started with a gray fixation after which one face stimulus (sized 2° × 2.71°) appeared for 200ms in one of the 9 experimental locations (see (**c**)), and then the fixation turned green indicating that a response about the emotional valence of the face was expected. The image presented in this figure is the same as in (**b**), a de-identified image from KDEF^23^. The background throughout the experiment was gray. See Methods for further details.

Images (front view portraits of 10 individuals (see Fig. 1a for example stimuli)) were selected from the NimStim image-set (5 female and 5 male actors, with the following de-identified database IDs: 01f, 02f, 06f, 07f, 18f, 21m, 22m, 28m, 33m, 37m; 02f and 22m were used only in practice session, the other 8 were used in the main experiment) each posing four expressions (fear, anger, neutral, happiness) with open- and closed-mouth versions of each emotion. Since in this study we were interested in testing whether our earlier results replicate with images from a different image set and of different individuals and to compare the findings of the two studies (thus comparing between the image sets), we made sure that the current study’s conditions precisely matched those of our previous study. To that end, in the current study we only analyzed emotion expressions of fear (open mouth), neutral (closed mouth), and happiness (open mouth) precisely as in our earlier study that used the KDEF image-set (see Fig. 1b for example stimuli). As in earlier studies^22,43^, photos were contrast-normalized and converted to grayscale using the ImageMagick open-source image processing tool (https://imagemagick.org/index.php) and scaled so that they occupy 2° × 2.71° of visual angle. The EyeLink Experiment Builder® software (Mississauga, Ontario, Canada: SR Research Ltd.) running on Windows 10 was used to present stimuli, record, and preprocess the data. The stimuli were displayed on an Eizo FG2421 24″ HD (1920 × 1080 pixels, 100 Hz) LCD monitor. Eye movements were recorded using an EyeLink 1000 DeskTop Mount (SR Research, Ontario, Canada) with a 35 mm lens, sampling rate of 500 Hz, and a chin rest located at 60 cm from the screen. The eye-tracking data, response times, and accuracy were exported using EyeLink Data Viewer version 3.2.1 (2018. Mississauga, Ontario, Canada: SR Research Ltd.) for further statistical analysis (see below).

### Procedures

Prior to the experimental session visual acuity was assessed using a logMAR chart that took a few minutes. The emotion valence categorization task was conducted in the same setup that our previous study was (darkened room and head stabilization by chin rest). The experiment was divided into runs of 216 trials each to allow participants to take breaks. Each run started with a standard eye-tracker built-in 5-point HV5 calibration procedure where the participant followed 5 consecutive points that appeared on the screen with her/his eyes while the eye tracker tracked their eyes, and this information was later used to extract eye position along the experimental time line. The experiment started with a short (up to 10 min) practice session, followed by sequence of runs of the main experiment that took 90–120 min depending on individual progress and duration of breaks participants needed between runs. Overall participation in the study took between 120 and 150 min.

As in our previous study, participants were instructed to fixate on a dark-gray fixation mark (0.3° diameter) in the center of the screen and initiate each trial by pressing the spacebar. Once the spacebar was pressed, a stimulus appeared for 200 ms in a randomly chosen location from the 9 parafoveal experimental locations (central, four in 2°, four in 4°, see Fig. 1c) which was followed by green fixation mark indicating that a response was expected (see Fig. 1d for a visualization of a trial timeline). Participants were required to report the valence of the just seen emotional face expression by pressing one of 3 keys (left for negative, down for neutral, right for positive). After the response was made, the green circle on the screen turned gray again until the participant initiated the next trial. Each of the 24 unique images [8 identities from NimStim each posing 3 emotions (of either negative, neutral, and positive valence)] appeared three times in each of the 9 locations during the experiment, resulting in 648 trials (8_ident.×_3_emotions×_3_reps.×_9_locations_; cf. 648 trials in our earlier study with 12_ident.×_3_emotions×_2_reps. ×_9_locations_ from the KDEF image set). The same experimental paradigm was used as in the single-trial experiment^22^ with the same image sizes, same 9 locations across visual field (see Fig. 1c), and same exposure duration (200 ms).

### Analysis

To verify that measured performance represented the specified visual field locations planned in our experiment, as in our earlier study here we also analyzed per-trial eye movements locations and filtered out trials with blinks or with distance of actual gaze position from screen center > 1°. No trials were excluded based on response times. Overall, in this study 22,318 trials were recorded by the eye tracker and in 15,392 trials (i.e., ~ 69%) fixation was kept. A total of 6926 trials (~ 31%) were excluded based on gaze position criteria (2347 of negative, 2291 of neutral and 2288 of positive valence) and these were distributed quite evenly across valences (*χ*^*2*^(2) = 1.005, *p* = 0.6; see Table S3 in Supplementary Material at https://osf.io/25fnw^38^ for descriptive summary of trials by fixations per condition in the current and earlier study, and Table S4 for additional results before excluding trials due to poor fixations). As in our previous experiment, here we calculated the mean categorization accuracy and mean response time (regardless of correctness; additional data and analyses of response times for correct responses only are available at https://osf.io/25fnw) for every participant separately per each experimental condition (for each valence per each eccentricity, see Table 1). Two 2-way repeated measures ANOVAs (on accuracy and on RT) with eccentricity and valence as factors were run as in our previous study. In this analysis there were 6 outliers as determined by studentized residual values. The outliers were kept in the analysis because they did not affect the results (see Table S5 in Supplementary Material for statistical analyses without outliers at https://osf.io/25fnw^38^). To test whether valence perception was associated across eccentricities as was done in our previous study, we correlated individual accuracies of each valence at 2° with those of each valence at 4°. We then examined for each of these correlations their non-directional statistical significance.

We examined potential across image-set differences using 3-way mixed ANOVAs (one for accuracy and one for reaction times) with eccentricity (0°, 2°, 4°) and emotional valence (positive, neutral, negative) as within participant factors and image-set (NimStim, KDEF) as between participants factor.

Greenhouse–Geisser corrections were applied whenever Mauchly's test of sphericity was significant. Post-hoc analyses were conducted with Bonferroni adjustment for multiple comparisons. All statistical analyses were performed with SPSS Statistics version 28.0.1.1 for Windows (IBM Corp. Released 2020).

Study Materials are available at the OSF repository at https://osf.io/25fnw.

## Results

**Figure 2:**
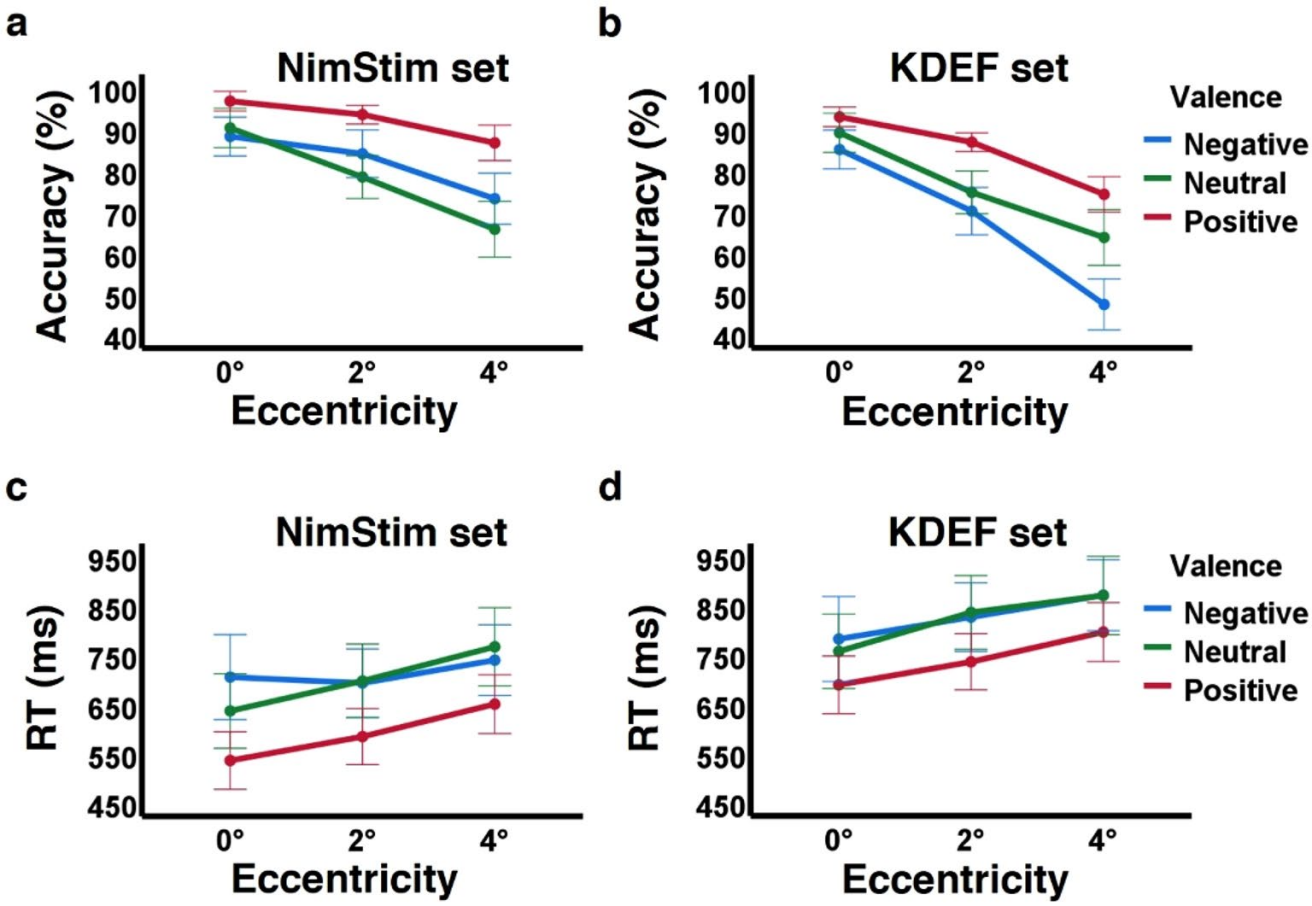
Perception of different facial valences are differentially modulated by eccentricity. Recognition performance of emotional valence decreased with growing eccentricity. Accuracy (**a**, **b**) decreased with growing eccentricity and RTs (**c**, **d**) became slower in the current study (**a**, **c**; with the NimStim image-set) and in the previous study^22^ (**b**, **d**; with the KDEF image-set). Positive valence in red, neutral in green, and negative in blue. Across the two studies positive valence was recognized better and faster and was less affected by eccentricity. It could also be seen that valence perception for NimStim stimuli was more accurate (cf. **a** and **b**) and faster (cf. **c** and **d**) than for KDEF stimuli. Accuracy-wise the positive vs. negative valence advantage evident at central vision increased with growing eccentricity (here tested up to 4° in the parafovea) and its magnitude was greater for KDEF than for NimStim stimuli. Error bars represent 2 standard errors.

To examine whether eccentricity affected valence categorization (descriptive summary in Table 1) we ran two separate two-way repeated measures ANOVAs (one on accuracy and one on RTs). As expected and in line with our previous findings^22^, performance accuracy declined significantly with growing eccentricity (*F*_(1.943, 53.731)_ = 102.179, *p* < 0.001, *ɛ* = 0.746, *ŋ*_*p*_^*2*^ = 0.739). This main effect of eccentricity on valence perception was evident by reduced accuracy of 6.4% at 2° relative to central vision (*p* < 0.001; from 91.5% ± 7.1% (SD) at 0° to 85.1% ± 8.5% (SD) at 2°), and an additional decline of 10.1% in accuracy at 4° (*p* < 0.001; 75.1% ± 11.8% (SD) at 4°, see Tables 1, 2, and Fig. 2a). This main effect of eccentricity on accuracy was also evident even before excluding any trials (*F*_(1.239, 44.589)_ = 113.962, *p* < 0.001, ε = 0.619, *ŋ*_*p*_^*2*^ = 0.760; see Table S4 in Supplementary Material for more details at https://osf.io/25fnw^38^). The eccentricity effect size we found was slightly smaller (*ŋ*_*p*_^*2*^ = 0.739 vs. estimated *ŋ*_*p*_^*2*^ = 0.853) but more significant than our a priori power analysis estimations (*p* < 0.001 vs. estimated *p* = 0.005, see Methods). This main effect of eccentricity was not a result of speed-accuracy tradeoff as reaction times were also slower in peripheral locations (significant effect of eccentricity on RT: *F*_(1.568, 56.459)_ = 21.447, *p* < 0.001, ε = 0.784, *ŋ*_*p*_^*2*^ = 0.373) with responses at 4° being slower on average by 93 ms relative to 0° (*p* < 0.001; from 625 ms ± 195 ms (SD) at 0° to 718 ms ± 184 ms (SD) at 4°, see Tables 1, 2, and Fig. 2c).

**Table 1:** Descriptive summary of accuracy and response times per condition (current (NimStim) vs. earlier (KDEF) study).

	Eccentricity	Valence			Mean per eccentricity
		Positive	Neutral	Negative	
Accuracy (% correct)					
NimStim (n = 37)	0°	96.6 ± 5.8	90.1 ± 15	88 ± 13.5	91.5 ± 7.1
	2°	93.3 ± 6.4	78.2 ± 14.1	83.8 ± 17.7	85.1 ± 8.5
	4°	86.4 ± 12.7	65.5 ± 21.3	72.9 ± 19.7	75 ± 11.8
	Mean per valence	92.1 ± 6.7	77.9 ± 14.9	81.6 ± 15.1	
KDEF (n = 37)	0°	92.8 ± 8.5	88.9 ± 13.9	84.9 ± 15.1	88.9 ± 8.7
	2°	86.7 ± 7.3	74.5 ± 17.3	69.9 ± 17.3	77 ± 9.2
	4°	74 ± 13.4	63.5 ± 20	47.2 ± 17.9	61.6 ± 10.3
	Mean per valence	84.5 ± 8	75.6 ± 15.2	67.3 ± 13.4	
RT (ms)					
NimStim (n = 37)	0°	535 ± 140	636 ± 224	704 ± 303	625 ± 195
	2°	584 ± 148	696 ± 208	692 ± 189	657 ± 169
	4°	649 ± 148	766 ± 239	739 ± 205	718 ± 184
	Mean per valence	589 ± 181	699 ± 207	712 ± 215	
KDEF (n = 37)	0°	688 ± 209	756 ± 235	781 ± 212	741 ± 197
	2°	734 ± 194	835 ± 245	825 ± 231	798 ± 213
	4°	795 ± 209	869 ± 243	869 ± 230	844 ± 216
	Mean per valence	739 ± 190	820 ± 225	825 ± 193	

Mean and standard deviation of accuracy (in % correct, top) and response times (in ms, of all trials included in the analysis, bottom) across participants of current study (n = 37, NimStim stimuli, above) and of previous study^22^ (n = 37, KDEF stimuli, below) by eccentricity (rows) and valence (columns). Summary across conditions on right and below. Additional data for response times for trials with correct responses only are available in Table S6 at Supplementary Material at https://osf.io/25fnw.

**Table 2:** Results of 2-way repeated measures ANOVAs of eccentricity and valence effects on accuracy and response times (for current (NimStim) and earlier (KDEF) study).

Factor	Current study n = 37; NimStim image set	Akselevich & Gilaie-Dotan, 2022 n = 37; KDEF image set
	Accuracy	Accuracy
Eccentricity	*F*_(1.493, 53.731)_ = 102.179, ***p*** < **.001**, *ε* = .746, *ŋ*_*p*_^*2*^ = .739	*F*_(1.453, 52.298)_ = 208.7, ***p* < .001**, *ε* = .726, *ŋ*_*p*_^*2*^ = .853
0°–2°	6.4% [4.1, 8.8], ***p* < .001**	11.9% [8.6, 15.2], ***p* < .001**
2°–4°	10.1% [7.6, 12.7]*, ****p* < .001**	15.5% [13.1, 17.8], ***p* < .001**
Valence	*F*_(1.411, 50.807)_ = 14.288, ***p* < .001**, *ε* = *.*706, *ŋ*_*p*_^*2*^ = .248	*F*_(2, 72)_ = 19.795, ***p* < .001**, *ŋ*_*p*_^*2*^ = .355
Positive–Neutral	14.2% [8.2, 20.2]*, ****p* < .001**	8.8% [2.4, 15.3], ***p* = .005**
Neutral–Negative	− 3.7% [− 12.5, 5.2], *p* = .92	8.3% [0.3, 16.4], ***p* = .041**
Eccentricity*Valence	*F*_(2.503, 90.114)_ = 5.673, ***p* = .002**, *ε* = .626, *ŋ*_*p*_^*2*^ = .136	*F*_(2.745, 98.824)_ = 9.082, ***p* < .001**, *ε* = .686, *ŋ*_*p*_^*2*^ = .201
Positive		
0°–2°	3.3% [0.03, 6.5], ***p* = .048**	6.1% [2.5, 9.8], ***p* < .001**
2°–4°	6.9% [3.2, 10.5], ***p* < .001**	12.7% [8.5, 16.9], ***p* < .001**
Neutral		
0°–2°	11.9% [6.7, 17.1], ***p* < .001**	14.5% [9.6, 19.4], ***p* < .001**
2°–4°	12.7% [8, 17.4], ***p* < .001**	11% [5.8, 16.2], ***p* < .001**
Negative		
0°–2°	4.2% [− 1.8, 10.2], *p* = .263	15% [7.5, 22.4], ***p* < .001**
2°–4°	10.9% [6.5, 15.4], ***p* < .001**	22.7% [17.7, 27.7], ***p* < .001**
	**RT**	**RT**
Eccentricity	*F*_(1.568, 56.459)_ = 21.447, ***p* < .001**, *ε* = .784, *ŋ*_*p*_^*2*^ = .373	*F*_(1.415, 50.95)_ = 13.666, ***p* < .001**, *ε* = *.*708, *ŋ*_*p*_^*2*^ = .275
0°–2°	− 32 ms [− 64, − 1], ***p* = .041**	− 57ms [− 95, − 18], ***p* = .002**
2°–4°	− 61 ms [− 92, − 30]*, ****p* < .001**	− 46ms [− 90, − 3]*, ****p* = .033**
Valence	*F*_(2, 72)_ = 22.217, ***p* < .001**, *ŋ*_*p*_^*2*^ = .382	*F*_(2, 72)_ = 21.519, ***p* < .001**, *ŋ*_*p*_^*2*^ = .374
Positive–Neutral	− 110 ms [− 158, − 62]*, ****p* < .001**	− 81ms [− 120, − 42]*, ****p* < .001**
Neutral–Negative	− 12 ms [− 63, 38], *p* = 1	− 5ms [− 42, 32]*, p* = 1
Eccentricity*Valence	*F*_(2.598, 93.528)_ = 2.809, *p* = .051, *ε* = .649, *ŋ*_*p*_^*2*^ = .072	*F*_(2.587, 93.119)_ = 0.505, *p* = .652, *ε* = .647, *ŋ*_*p*_^*2*^ = .014

Results for accuracy above, for RTs below, for current study (with NimStim stimuli) on left, for our earlier study^22^ (with KDEF stimuli) for comparison on right. Main effects with post-hoc analyses (with Bonferroni corrections) presented with mean differences and 95% confidence interval [lower limit, upper limit] followed by interaction results (see Results for additional analysis related to positive vs negative valence differences). Significant results in bold. In both studies significant effects of eccentricity and valence on accuracy and RT were found as well as a significant interaction for accuracy only. Additional RT analyses for trials with correct responses only are available in Table S7 at https://osf.io/25fnw.

We also examined whether valence modulated performance (as in our previous findings) and found a significant effect of valence on accuracy (*F*_(1.411, 50.807)_ = 14.288, *p* < 0.001, ε = 0.706, *ŋ*_*p*_^*2*^ = 0.284). This effect of valence on accuracy was evident by 14.2% higher accuracy (*p* < 0.001) for positive valence (92.1% ± 6.7% (SD)) relative to neutral valence (77.9% ± 14.9% (SD)) and no difference (*p* = 0.92) between accuracy for neutral and negative valence (81.6% ± 15.1% (SD); compare with KDEF where we found that accuracy for neutral was higher than that of negative valence, see Table 2). This main effect of valence on accuracy was significant even before trial exclusion (*F*_(1.435, 51.666)_ = 12.625, *p* < 0.001, *ε* = 0.718, *ŋ*_*p*_^*2*^ = 0.260; see Table S4 at https://osf.io/25fnw for more details^38^). This valence effect size was also slightly smaller (*ŋ*_*p*_^*2*^ = 0.284 vs. estimated *ŋ*_*p*_^*2*^ = 0.355) but more significant than we estimated in our power analysis (*p* < 0.001 vs. estimated *p* = 0.005) with positive valence showing higher accuracy in each eccentricity (*p* < 0.001) in line with our recent findings (see Tables 1, 2 and Fig. 2b). We found a significant interaction between eccentricity and valence on accuracy (*F*_(2.503, 90.114)_ = 5.673, *p* = 0.002, *ε* = 0.626, *ŋ*_*p*_^*2*^ = 0.136) that reflected smaller effect of eccentricity on positive valence accuracy than that found for negative and neutral valence accuracy (positive valence accuracy decreased by 10.1% at 4° relative to 0° while neutral valence accuracy by 24.5% and negative valence accuracy by 15.1%; see Fig. 2a). This was also found in our earlier study with the KDEF images where positive valence accuracy was less affected by eccentricity than neutral or negative valence accuracy (positive valence accuracy decreased by 18.8% at 4° relative to 0°, while neutral and negative valence accuracies by 25.4% and 37.7% respectively; see Fig. 2b). A direct comparison between positive and negative valence accuracies revealed that positive valence accuracy was higher than negative valence accuracy by 10.53% (95% confidence interval of [5.1, 15.9], p < 0.001). A more detailed analysis examining this difference by eccentricity revealed that at central vision positive valence accuracy was higher than negative valence accuracy by 8.6% (95% confidence interval of [2.9, 14.3], p = 0.002), at 2° by 9.5% (95% confidence interval of [2.6, 16.3], p = 0.004), and at 4° by 13.5% (95% confidence interval of [6.1, 21.0], p < 0.001; similar analysis for KDEF study are available below). Furthermore, this interaction between valence and eccentricity was also significant even before any trial was excluded (*F*_(2.228, 80.21)_ = 13.242, *p* < 0.001, *ε* = 0.557, *ŋ*_*p*_^*2*^ = 0.269; see Table S4 at https://osf.io/25fnw for more details^38^). As can be seen in Fig. 2c, the effect of valence on performance was also not a result of speed-accuracy tradeoff as the significant effect of valence on RTs (*F*_(2, 72)_ = 22.217, *p* < 0.001, *ŋ*_*p*_^*2*^ = 0.382) was evident by fastest responses for positive valence (*p* < 0.001) and no significant response time difference between neutral and negative valences (*p* = 1). No interaction between eccentricity and valence was found on RTs (*F*_(2.598, 93.528)_ = 2.809, *p* = 0.051, *ε* = 0.649, *ŋ*_*p*_^*2*^ = 0.072). Additional RT analysis taking into account only trials with correct responses was consistent with the analyses for all RTs and these data and findings are available at https://osf.io/25fnw (see Tables S6 and S7 at Akselevich&Gilaie-Dotan_replication_SuppMat.docx). In sum, our results replicated our earlier findings for an effect of eccentricity and valence on accuracy and RTs, with positive valence showing highest performance across eccentricities, measures and studies (Fig. 2).

### Cross-conditions analyses

**Figure 3:**
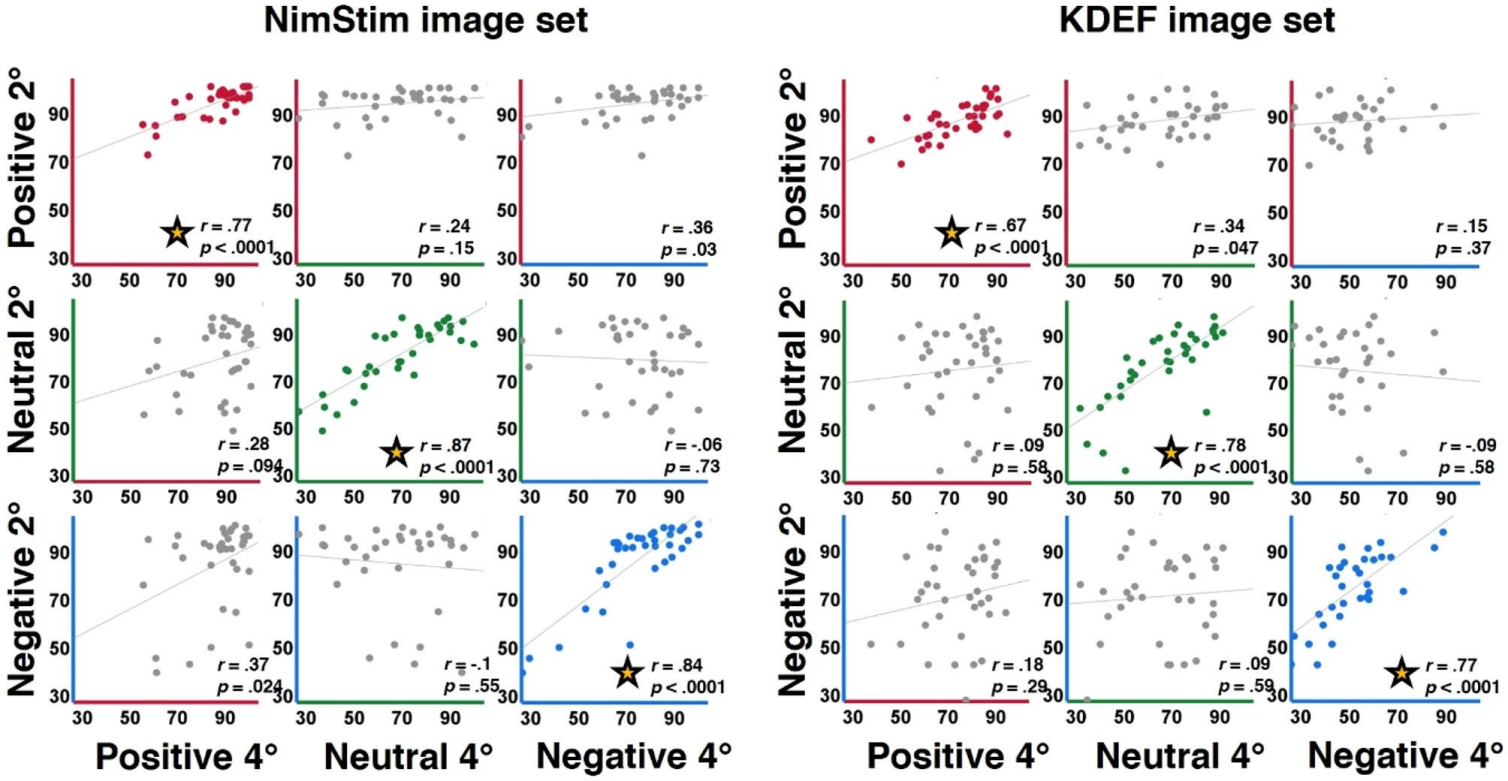
Within-valence but not between-valence performance is correlated across eccentricities. Scatterplots from current study (left), from our earlier study^22^ (right). Each scatterplot represents a correlation between parafoveal accuracies of each valence at 2° (on the y-axis by valence) to each valence at 4° (on the x-axis by valence). Within each scatterplot, each point represents performance of one participant. R values represent Pearson correlation coefficients, and *p* indicates non-corrected correlation significance. Note that only within-emotion correlations (positive 2° to positive 4°, neutral 2° to neutral 4°, negative 2° to negative 4°, presented on the diagonals) were significant (surviving multiple comparisons correction, in bold, Bonferroni correction threshold at *p* = .0056) and are denoted by asterisks, and these results were consistent across the two studies.

In our earlier study we found within-valence but not across-valence perception correlations in the parafovea. Here, we followed the same correlation analyses (see Fig. 3) finding that the results were consistent with our earlier findings. Specifically, we found significant correlations within emotional valence (2° to 4° within-valence performance correlation for positive: *r*(35) = 0.77, *p* < 0.0001, for neutral: *r*(35) = 0.87, *p* < 0.0001, and for negative: *r*(35) = 0.84, *p* < 0.0001, all surviving multiple comparisons correction) but not between-emotional valence (2°–4° across-valence performance correlation: none surviving multiple comparisons (n = 9) correction, see Fig. 3). As in our previous study, similar correlation analyses with RTs revealed that all across-eccentricity RT correlations were significant (all *r*’s ≥ 0.65, all *p*’s < 0.001) and this was also the case when only correct responses were taken into account (all *r*’s ≥ 0.51, all *p*’s < 0.001). These results may indicate that RTs reflect individuated response speed behavior and not necessarily perceptual aspects measured here in the study. These cross-condition correlational analyses replicated our earlier findings and may suggest that perception of different valences may be supported by dissociated mechanisms.

### Controlling for potential confounding factors

As in our previous study, here too, we were interested to investigate whether the heightened performance for positive valence relative to negative valence resulted from potential response bias towards “positive valence” responses and/or refraining from responding “negative valence”. Frequency distribution of incorrect responses per eccentricity for each valence condition (see Table 3 for details) were similar to the findings in our previous study^22^. First, “positive valence” was not the most frequent incorrect response (“false alarms”) in any condition. Critically, in the neutral valence condition when participants responded incorrectly, the most frequent choice was towards “negative” (75% of errors; *χ*^*2*^ (1) = 350.1, *p* < 10^−35^) and not “positive”, indicating that there was no response bias towards “positive” or response bias refraining from “negative”. Thus, we can rule out the possibility that the results were driven by a response bias towards positive valence or refraining from choosing negative valence. Note however that this analysis cannot rule out the possibility that there may be a response bias towards “negative” (and/or refraining from responding “positive”) and if that is the case it indicates that the difference we found in how eccentricity modulates positive vs negative valence accuracies may even be larger than we measured.

**Table 3:** Frequency of incorrect responses by eccentricity and valence.

Conditions		Incorrect responses			Sum
Eccentricity	Stimulus valence	Positive	Neutral	Negative	
0º	Positive	–	9	**10**	19
	Neutral	17	–	**42**	59
	Negative	23	**54**	–	77
2º	Positive	–	71	**85**	156
	Neutral	110	–	**423**	533
	Negative	109	**197**	–	306
4º	Positive	–	146	**148**	294
	Neutral	218	–	**577**	795
	Negative	223	**319**	–	542
Sum		700	796	**1285**	2781

Frequency of incorrect responses per eccentricity for each valence. In bold the most frequent type of error per condition. Analysis based on trials with fixation kept within 1° from central vision. Note that “positive valence” response was not the most frequent choice in any condition, suggesting that the results are not driven by a “positive valence” response bias. Furthermore, in 4 of the 6 parafoveal conditions the majority of the incorrect responses were toward “negative valence” choice, providing support that the results are not a consequence of refraining from responding “negative valence” in the parafovea.

### Comparing NimStim to KDEF results

**Figure 4:**
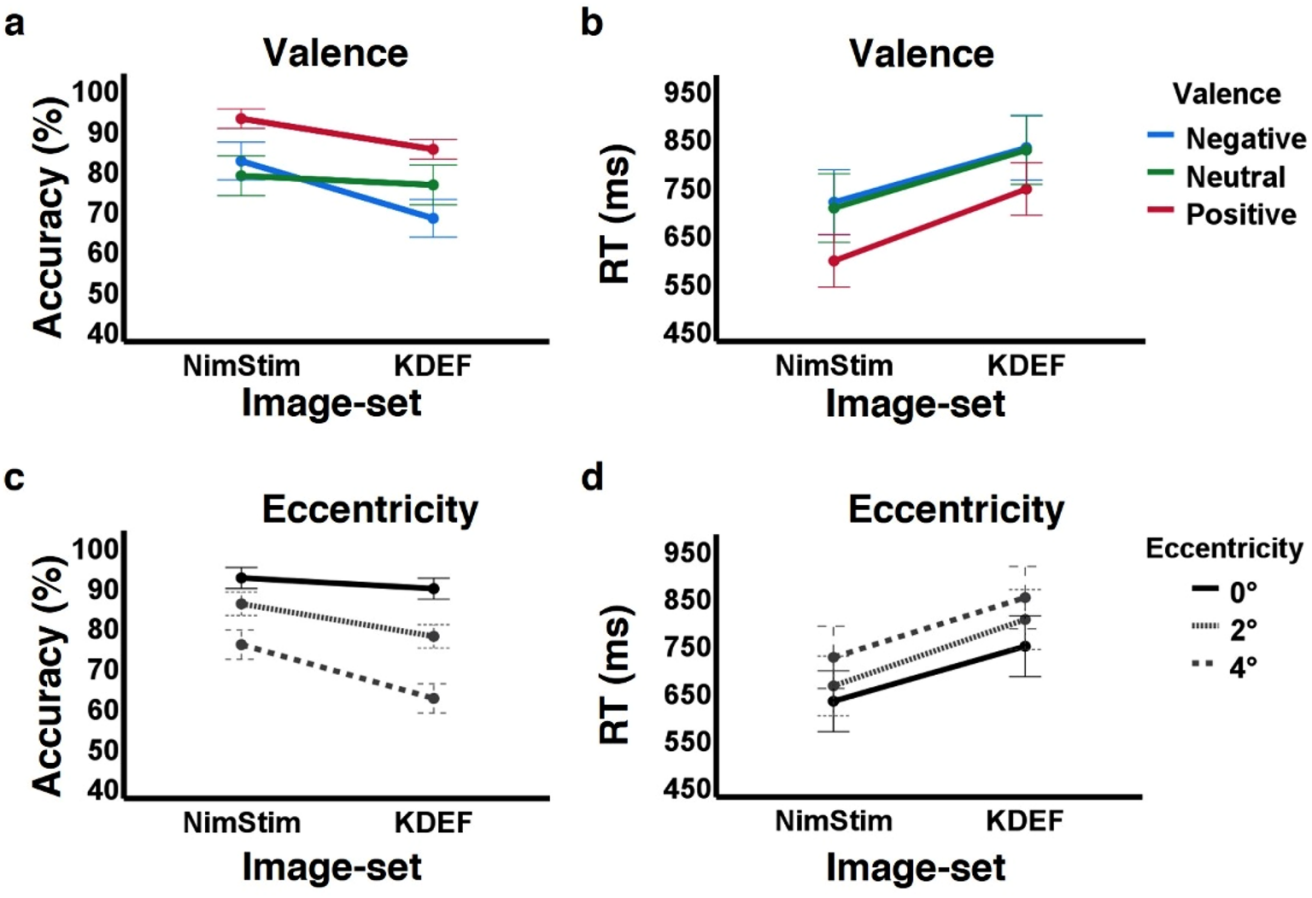
Main effects replicated across studies but image-set influenced performance. Comparison of accuracy (in % correct) and reaction times (in ms) by image-sets. In each panel results for current study with NimStim stimuli on left (n = 37) and for our earlier study^22^ with KDEF stimuli on right (n = 37). Recognition accuracy by valence (**a**) or by eccentricity (**c**); RTs by valence (**b**) or by eccentricity (**d**). Note that higher accuracies and faster responses were found for the NimStim stimuli. Error bars represent 2 standard errors.

The main findings from our previous study with the KDEF image-set^22^ replicated in the current study with the NimStim image-set. As the magnitude of the effect appeared to be smaller with the NimStim stimuli (higher overall accuracy and faster RTs than those found in the KDEF study) we directly compared the results of the two studies running two separate 3-way mixed design ANOVAs (one for accuracy, one for RTs) with image-set (NimStim vs KDEF, between participants), eccentricity (within participants), and valence (within participants) as factors. The main effects of eccentricity, valence and their interaction on recognition accuracy were all significant (all *p’*s < 0.001) and consistently replicated the results we obtained in each of the studies separately. In addition, the main effect of image-set showed that accuracy with KDEF images (75.8% ± 8.1% (SD)) was 8% lower (*p* < 0.001, 95% CI [− 11.9, − 4.2]) compared to accuracy with the NimStim images (83.9% ± 8.4% (SD)). Accuracy performance differences between the image sets were evident only at parafoveal eccentricities (see eccentricity by image-set interaction in Table 4). In addition, accuracy differences between the image-sets were evident for positive (*p* < 0.001) and negative (*p* < 0.001) e (*p* = 0.517; see valence by image-set interaction in Table 4 and Fig. 4).

**Table 4:** Statistical analysis of the effects of image set (NimStim vs KDEF) on valence perception in the parafovea.

Factor		Accuracy	RT
Image-set		*F*_(1, 72)_ = 17.491, ***p*** < **.001**, *ŋ*_*p*_^*2*^ = .195	*F*_(1, 72)_ = 8.674, ***p* = .004**, *ŋ*_*p*_^*2*^ = .108
NimStim, KDEF		8% [4.2, 11.9], ***p* < .001**	− 128 ms [− 214, − 41], ***p* = .004**
Eccentricity*Image-set		*F*_(1.50, 108.21)_ = 18.155, ***p* < .001**, *ε* = .751, *ŋ*_*p*_^*2*^ = .201	*F*_(1.47, 105.95)_ = .497, *p* = .553, *ε* = .736, *ŋ*_*p*_^*2*^ = .007
0°	NimStim, KDEF	2.7% [− 1, 6.3],* p* = .153	− 116 ms [− 207, − 26], ***p* = .013**
2°	NimStim, KDEF	8.1% [4, 12.2],*** p* < .001**	− 141 ms [− 230, − 52],*** p* = .002**
4°	NimStim, KDEF	13.4% [8.3, 18.5],*** p* < .001**	− 126 ms [− 219, − 34],*** p* = .008**
Valence*Image-set		*F*_(1.575, 113.368)_ = 4.78, ***p* = .016**, *ε* = .787, *ŋ*_*p*_^*2*^ = .062	*F*_(2, 144)_ = 1.176, *p* = .312, *ŋ*_*p*_^*2*^ = .016
Positive	NimStim, KDEF	7.6% [4.2, 11],*** p* < .001**	− 150 ms [− 226, − 73],*** p* < .001**
Neutral	NimStim, KDEF	2.3% [− 4.7, 9.2],* p* = .517	− 121 ms [− 221, − 20], ***p* = .019**
Negative	NimStim, KDEF	14.2% [7.6, 20.9],*** p* < .001**	− 113 ms [− 208, − 19],*** p* = .020**
Eccentricity*Valence *Image-set		*F*_(2.7, 194.92)_ = 5.841, ***p* < .001**, *ε* = .677, *ŋ*_*p*_^*2*^ = .075	*F*_(2.7, 194.93)_ = .92, *p* = .424, *ε* = .677, *ŋ*_*p*_^*2*^ = .013
0°			
Positive	NimStim, KDEF	3.8% [0.4, 7.1],*** p* = .029**	
Neutral	NimStim, KDEF	1.1% [− 5.6, 7.8], *p* = .742	
Negative	NimStim, KDEF	3.1% [− 3.5, 9.8],* p* = .352	
2°			
Positive	NimStim, KDEF	6.6% [3.4, 9.8], ***p* < 0.001**	
Neutral	NimStim, KDEF	3.7% [− 3.7, 11],* p* = .317	
Negative	NimStim, KDEF	13.9% [5.8, 22], ***p* = .001**	
4°			
Positive	NimStim, KDEF	12.5% [6.4, 18.5],*** p* < .001**	
Neutral	NimStim, KDEF	2% [− 7.6, 11.6],* p* = .677	
Negative	NimStim, KDEF	25.7% [17, 34.4],*** p* < .001**	

Partial results of 3-way mixed ANOVAs with image-set (NimStim (current study) vs earlier study^22^ with KDEF images), eccentricity, and valence effects on accuracy (left) and on response times (RT, right) detailing effect of image set and interactions with image set. Main effects of and interaction between eccentricity and valence were significant as was the case in each experiment separately. Bonferroni corrections applied in post-hoc analyses, mean differences reported with 95% confidence interval [lower limit, upper limit]. Significant results in bold. See also Fig. 4.

We also directly compared between positive and negative valence accuracies (as performed for NimStim study results, see above) and this comparison revealed that positive valence accuracy was higher than negative valence accuracy by 17.2% (95% confidence interval of [11.3, 23.0], p < 0.001). A more detailed analysis examining this difference by eccentricity revealed that at central vision positive valence accuracy was higher than negative valence accuracy by 7.9% (95% confidence interval of [1.8, 14.1], p = 0.002), at 2° by 16.8% (95% confidence interval of [9.9, 23.6], p < 0.001), and at 4° by 26.8% (95% confidence interval of [17.5, 36.0], p < 0.001). This analysis suggests that at central vision positive valence accuracy was higher than negative valence accuracy in a similar manner across the two image-sets, but at parafoveal vision the differences between positive and negative accuracies became bigger with KDEF (see also Fig. 2a,b).

The higher accuracy with the NimStim stimuli was not driven by speed-accuracy tradeoff since responses were significantly faster with the NimStim stimuli (mean difference of 128 ms, *p* = 0.004, 95% CI [41, 214]) and these faster response times for NimStim stimuli were evident in all eccentricities and for each valence (see rightmost column in Table 4 and Fig. 4b,d).

To examine the possibility that the performance differences between the image sets resulted from between image-set differences in the visual angle that the face stimuli occupied, we directly measured the visual angle that each face occupied (as a measure of face size we measured face width from one cheekbone to another) for every stimulus used in each of the image-sets. We found that NimStim face widths (1.397° ± 0.056° (SD)) were significantly wider (mean difference of 0.016° (95% CI [0.13, 0.19]), *t*(59) =  − 10.762, *p* < 0.000001, 2-tailed) than the KDEF face widths (1.24° ± 0.057° (SD)), indicating that face sizes in NimStim images were significantly larger than those in KDEF images. Thus, these differences may partially contribute to the difference in performance found between the two image-sets.

To summarize, valence categorization performance for the NimStim image-set was higher than that of the KDEF image-set and this was evident in higher accuracy and faster response times.

## Discussion

In this study we assessed whether our recent findings of eccentricity modulating valence perception are replicated with different stimuli in a new cohort. Here, as in our recent study, we found that valence perception was modulated by eccentricity, that positive valence was affected the least, that the positive valence advantage extended to the parafovea (up to 4°), and that within-valence (but not between-valence) performance was predictive across the parafovea. The replication of our earlier results was robust and direct between-study comparison revealed that the effects were milder in magnitude with the new image set (NimStim^25^) than those found with the previous image set (KDEF^23^).

### Eccentricity modulates performance in a valence-specific manner

It is well known that vision degrades with eccentricity (“eccentricity effect”^44^) but our findings that different valences are affected differently by eccentricity cannot be fully attributed to the cortical magnification factor (CMF^45–47^) that we have not compensated for (similarly to other high-level vision studies using faces^39^). While central vision is over-represented in the cortical surface relative to its proportion on the retina^48–50^, the CMF can account (at least to some extent) for the overall eccentricity-based reductions^51^ but not to the differential valence-specific effects we observed. Earlier studies with centrally presented facial expressions show that the perception of happy expressions is more accurate^13,52^ and this has been suggested to result from the higher frequency of happy expressions that people encounter^53–55^. It has also been shown that in “face pareidolia”—a phenomenon where the visual system interprets a subset of non-face stimuli as face-like, people tend to attribute emotional states to these illusory faces with happy expressions more likely than other expressions to be perceived^56^. In addition, it has also been suggested that positive valence expressions are more “uniform” (typically with a smile) while negative valence emotions are more diverse, which could lead to improved familiarity with positive rather than with negative valence expressions of emotion. However, these are unlikely to explain the interaction between valence and eccentricity we found across both studies, which indicates that it is not merely an amplification of positive valence performance but rather modulation guided by eccentricity. The fact that we found in both of our studies within-valence associations across eccentricities (e.g. positive valence accuracy at 2° correlated with positive valence accuracy at 4°) and no clear support for across-valence associations (e.g. positive valence accuracy at 2° not significantly correlated with negative or neutral valence accuracy at 4°) further supports the idea that there may be additional valence-specific mechanisms that are differentially affected by eccentricity. One possible explanation that may explain why there is enhanced performance for positive expressions in the parafovea could be that positive faces appearance may be more homogeneous and more salient than expressions of neutral or negative valence. One study by Calvo and Nummenmaa (2008) investigating facial expression perception in the parafovea^21^ supports this suggestion where they found that performance for positive valence in a visual search task in the parafovea was significantly better for positive expressions. Another study investigating facial expression perception at central vision indicates that the mouth region attracts attention only for positive expressions while the eye region is attended for neutral and negative expressions and they suggest that these differences could be driven by low level image features^57^. Another study examining these face features contributions to facial emotion perception in central and peripheral vision^20^ corroborates these results for central and peripheral vision (up to 6°) finding that even in peripheral vision the sensitivity to happy (positive valence) expressions relies predominantly on the mouth region whereas sensitivity to fearful (negative valence) expressions relies mainly on the eye region. These studies together with additional findings showing heightened performance for happy faces in peripheral vision^19,20,22^ suggest that at growing eccentricities when visual information is reduced the information available for positive valence expressions may be easier to distinguish from the other valences leading to more robust cues for happy faces in the periphery which may make them easier to identify. We cannot rule out a possibility that there may be a perceptual bias towards happy (positive valence) faces (i.e. “happy bias”) given the significant differences we found in performance in the parafovea in both of our studies as well as additional findings predominantly in central but also in peripheral vision. Importantly, given the response bias analysis we carried out that ruled out the possibility that this reflects a response bias towards positive valence (happy faces), if such a perceptual bias towards happy faces exists it means that it may even be larger in magnitude than what we measured here. Our studies indicate that if such a “happy bias” exists it extends to parafoveal vision and may even grow with eccentricity as our data suggest (positive valence accuracy was higher than negative valence accuracy and this difference grew with eccentricity). Another plausible explanation that may underlie the enhanced performance to positive expressions in parafoveal vision could be related to enhanced developmental experience with positive expressions. For example, more positive valence as smiles during early childhood^54,58,59^ could perhaps lead to expansion of visual field sensitivity to happy faces while other emotions that are less prevalent during this developmental period may be left with narrower visual-field sensitivity. Indeed, a study demonstrates that by the age of 5 months babies may already be capable of distinguishing between different types of smiles^60^. Furthermore, Bornstein and colleagues^58^ found that infants of non-depressed mothers discriminated between neutral and smiling expressions, while infants of clinically depressed mothers did not exhibit this ability despite no difference in their initial attention to faces. The researchers of that study pointed out that disparities in the ability to discern emotions might arise from differences in early experience between these two groups. They also suggest that since clinically depressed mothers tend to display flatter affect and less positive emotions their infants are exposed to fewer smiling facial expressions. Our results are based on the general adult population and as we did not obtain information about childhood experience^58,61^ from our participants, we cannot make direct inferences about their influence. Thus, while we repeatedly found that different valences are differentially modulated by eccentricity in the parafovea, the underlying mechanisms are yet unclear.

### Image-set influences on parafoveal performance

While our recent findings^22^ were replicated in the current study, we also found that performance in the current study using the NimStim stimuli was overall significantly better (higher accuracy and faster response times) relative to our earlier results reported with KDEF. It is important to note that image-set influences were not uniform across eccentricities (higher accuracy in the NimStim study found only for peripheral conditions) or across valences (higher accuracy for NimStim only found for positive and negative valence, not for neutral). There are a few potential factors that may account for or contribute to these between image-set performance differences. First, there are indications that the NimStim images convey expressions more intensely compared to the KDEF images^24^ and this by itself could be informative^62^ not only at central vision but also in the parafovea, leading to enhanced performance (higher accuracy and faster RTs; this may also perhaps explain why negative performance was closer to that of neutral in NimStim and not in KDEF). Second, the area that the faces occupied within the KDEF stimuli was slightly smaller than the area the faces within the NimStim stimuli occupied (see Results). Such a difference may partially influence performance. Third, in the current study each image was repeated three times in each location across the experiment, while in our earlier study^22^ each image was repeated only twice. This enhanced exposure to each image (50% more) in each location may have led to enhanced familiarity with the images but is unlikely to have influenced specific valence performance since trial order was randomized. Importantly, while these and other factors may explain the performance differences between the image-sets, our main findings of eccentricity differentially modulating performance according to valence still hold here with the NimStim stimuli across identities and intensity of expressed emotions^24,63^, and this is important given that a mere change of stimulus set can significantly influence emotion-based judgements^64^.

### Main effects replicated across studies

While there are differences between the magnitude of the effects we found and that found in our earlier study, it is of great importance that all the results have been replicated, especially given the replication crisis in the field of cognitive psychology^35–37^. In the field of emotional perception some studies suggest that positive valence perception is superior to negative valence perception^17,27^ while other studies suggest the opposite^30,65^. The positive or negative valence superiority may be questionable given that opposing results may even be found within a study^64^. These between-study differences could reflect experimental design differences requiring different strategies and different levels of processing^18^. Several methodological factors in experimental designs may contribute to these inconsistent findings. One of these factors relates to the stimulus sets used in studies that often employ qualitatively different types of stimuli, for example schematic faces or drawings^30,65,66^, full face photos or cropped face parts^67^, or videos of dynamic expressions^62^. Additional factor relates to stimulus presentation duration that ranges across studies from very short durations (10–50 ms^68^; 200 ms^69^) through several seconds (3s^14^) to unlimited exposure duration^67,70^. Another potential factor is the experimental task that varies across studies (e.g., recognition, detection, discrimination^71^ and/or different emotional categories^64,72^). While these and other factors may contribute to the inconsistencies in the field, here we aimed to test the replication of our earlier findings while modulating image-set (using another stimulus image-set and with different identities) and keeping other factors consistent with our recent paradigm. Importantly, as is recommended to substantiate transparency given the replication crisis^36,37^, we followed here all the suggested recommendations for authors^36^ that were proposed to overcome potential pitfalls and serve as an effective disclosure-based solution. As we described in the Methods (see also supplementary material at https://osf.io/25fnw^38^), we followed a power analysis and decided in advance on the number of participants to be recruited. We collected for each cell > 20 observations (n = 37), we described all the variables collected in our study (as those in our earlier study^22^), we reported all our experimental conditions and findings, and we reported the statistical analysis with and without applying the exclusion criteria (i.e. non-fixated trials), finding that the effects of eccentricity and valence and their interactions were robust and highly significant even before exclusion criteria were applied (as we did not have any covariates, these were not reported). Having adopted these practices, we found that the main effects of eccentricity and valence type on valence recognition were robustly reproduced. These results further substantiate the finding that eccentricity differentially modulates perception of different valences for static images presented for brief exposures. Furthermore, in order to genuinely measure parafoveal and peripheral vision one must ensure that eye movements (actual locus of fixation) are being monitored.

### Constraints on generality

It is still not possible to rule out several factors that may limit further generalization of our findings. For example, the expressions in both image-sets are based on static photos of posed and not spontaneous (genuine) expressions (which are typically dynamic in daily behavior) which could influence perception^62,73^. Our lab-based investigations thus far only employed one positive and one negative emotion per valence while the range of real positive and real negative emotions is much broader. Therefore, we cannot also rule out the possibility that our results reflect differences between two specific emotional categories (fearful vs happy) and may not generalize to additional emotional categories within each valence. In a similar manner we cannot determine whether our results related to parafoveal valence categorization will fully generalize to expression categorization in the parafovea (expression categorization was tested in the parafovea with a different paradigm with KDEF images^20^ finding happy faces best categorized in peripheral vision, but these results were not compared to performance with NimStim stimuli). In our studies people were exposed to very brief presentations of emotional facial expressions (200ms) which may tap into very initial stages of valence processing and may not fully reflect the complete timeline of everyday valence processing that can last seconds or longer. Furthermore, since our study aimed to test the replicability of the results obtained in our earlier study, it was important that our current study would follow similar cohort specifications. Specifically, while the only inclusion criteria were age range (18–35 years) and normative or corrected to normative vision, as in our pervious study, here too our cohort was limited to young adults from middle-to-high socioeconomic background, mostly educated (see demographics) and in a western industrialized democratic society since these are the only people that enrolled. Therefore, generalization to other populations is yet to be established^14,52,62,74–78^. However, even given all these potential limitations and possibly additional ones, we were able to successfully replicate our earlier findings that eccentricity modulates valence perception and affects negative and positive valence in a differential manner. These results substantiate our earlier findings and reveal that differences between facial emotional image-sets are not confined to central vision and extend to parafoveal vision (potentially in a differential manner).

## Conclusions

In this study we found that valence perception of facial expressions of emotions in the parafovea (up to 4°) was affected by eccentricity, that eccentricity modulated different valences in a different manner, and that there were significant differences in the magnitude of these observed effects that are related to the image-sets used. Specifically, we found that while valence categorization was affected by eccentricity for each and every valence, positive valence was affected the least and negative to a much greater extent. We found that for each valence its parafoveal valence categorization at 2° predicated its performance at 4° (but not that of other valences). When comparing performance according to the image sets employed in our studies, performance with NimStim stimuli was higher than performance with KDEF images, and interestingly as parafoveal perception of valence became more difficult (at 4° with KDEF images) the difference between positive and negative valence categorization became greater. Together, our results indicate that eccentricity and image-set are two dominant factors that modulate valence perception in the parafovea.

## Data Availability

Supplementary anonymized data generated and/or analyzed during the current study are available as open data in the Open Science Framework repository at https://osf.io/25fnw.
